# A New Method of Mummification of the Pulp

**Published:** 1899-10

**Authors:** 

**Affiliations:** Prague, Bohemia.


ARTICLE VI.

### A NEW METHOD OF MUMMIFICATION OF THE PULP.

PROFESSOR BOENNECKEN, M. D., D. D. S., PRAGUE, BOHEMIA.

The following report is a brief abstract of two original communications upon modern methods in the treatment of diseased pulps that have appeared in the "Oesterrichische Ungarische Vierteljahrs-schrift fur Zahnheilkunde," Vienna, January, 1898, and January, 1899. In these papers I have given a report of experiments made by me in the course of the last four years upon the subject of mummifying the pulp after its devitalization by arsenic. Having reached in the beginning of this year a record of a thousand successful case of acute or chronic pulpitis, all treated in the same way without removing the root portions of the pulp, I venture to give the readers of the "Dental Cosmos" a brief description of my method.

Every practitioner knows that in a great many cases of pulpitis in molars or bicuspids, mechanical difficulties will make the total extirpation of the devitalized pulp a matter of impossibility. In another great percentage of cases we cannot remove the pulp because we dare not inflict the pain upon our patient. Indeed, this little operation is very often a greater torture than the extraction of the teeth itself. I do not know whether American patients can stand a greater amount of pain in the dental chair than European ones, but I can assure you that in our country, and especially in the large capitals of our continent, the dental parlors are full of neurasthenic and hysteric patients who cannot endure the perfect removal of a devitalized molar pulp without paying for it with a few days' suffering from severe nervous prostration.

For this kind of invalids the treatment which I am going to describe seems to me almost a necessity. I may add that busy dentists, especially those colleagues who are engaged in "praxis pauperum," very often cannot find the necessary time for a careful cleaning and filling of the root-canals after the old method, that we have been taught in the college, On the other hand, a tooth with fragments of a semi-devitalized pulp left in the canals may be for a certain period of time a source ef great annoyance to the bearer. Until the last trace of living pulp-tissue gets perfectly mummified, which may require a couple of months, or even years, the tooth will answer to every cold or hot drink, and will not allow a normal mastication. If, however, the pulp-tissue which has been left in the canals was already infected with bacteria, an apical abscess is certain to appear sooner or later. Therefore, for all cases when we are not either able or willing to perform the total extirpation of the pulp we need some therapeutic agent that will not only perfectly and rapidly devitalize, but also permanently sterilize the fragments of the pulp.

Miller, in his admirable text-book of operative dentistry, recommends little tablets of murmuric chlorid 0.003 gram and thymod 0.005 gram to be crushed by means of amalgam plugger in the pulp-chamber after the amputation of the crown portion of the pulp. I have tried this method in about a hundred cases, with the result that these teeth, after a short period of pericemental irritation, became perfectly healthy and quiet. The irritation lasted from two hours to three days. Afterward those molars worked like healthy teeth, and never gave any more trouble. The only objection to this method, which is excellent for clinical practice, is the greenish discoloration of the crown, therefore its use ought to be restricted to molar teeth.

For the last four years I have been experimenting in the same direction with different preparations of formaldehyd. Formaldehyd, or formalin in its aqueous solution, has two qualities which make it extremely valuable for our purpose of mummifying the devitalized pulp. It is a powerful sterilizer, and it coagulates the albumen of the living tissue. Indeed, it hardens the protoplasm so quickly that one can change the soft tissue of a pulp into the consistence of leather by half an hour's application of formalin. Therefore, it has of late been widely used for the conservation of anatomical and histological specimens.

The plan of my experiments was to sterilize and to coagulate the root portions of the pulp with farmalin; in other words, to harden the pulp like a microscopical specimen, and. as formalin produces little or no shrinking of the tissue, to change the semi-devitalized and infected stumps of the pulp into totally devitalized and aseptic root fillings. If began by washing out the pulp chamber with the concentrated solution of formaldehyd and sealing in the chamber a piece of cotton wool, saturated with forty per cent. formalin. All the patients treated in this way suffered between half an hour and ten hours from a severe burning toothache. The stage of irritation being once over, the teeth become comfortable, and patients soon forget that they ever had trouble with them. I discovered that the painful reaction of the semi devitalized pulp stumps was due to the high concentration of formalin. By gradually diminishing the strength of formalin and mixing the formaldehyd with cocain and thymol to a thick paste, I arrived at a method which I have tested now in more than a thousand cases. I now treat every case of inflammation of the pulp in molars or bicuspids, as follows :

Twenty-four to forty eight hours after the application of arsenic I remove the crown portion of the pulp (the so-called amputation of the pulp) with a large, sharp bur, using the warm water syringe for cleaning out all the *debris* of the cavity and the pulp chamber. This little operation is almost in every case quite painless. Then the rubber-dam is applied and the pulp-chamber thoroughly washed out with a ten per cent. solution of formaldehyd (one volume of the forty per cent. formalin to three volumes of water). I call this the "formol-bath" of the pulp. Then I fill the pulp-chamber with my formaldehyl paste, cover with oxyphosphate, and put in the permanent filling. The tooth is always finished in one sitting.

Two years ago I published the following formula for the formaldehyd paste :

℞—Cocain,
Thymoli, aa 1, 0;
Misce exactissime terendo.
Adde sol. formaldehydi aquos (40%), gtt. x;
Zinc oxid, 2, 0.
Fiat pasta.

But a formaldehyl paste exposed to air in an open bottle does not waste very well. The formaldehyd soon evaporates and disintegrates; besides, the paste soon loses the proper consistence—it is either too thick or too thin to be handy for dental purposes. Therefore, I gave an order to Mr. van Tongel, an eminent chemist of Prague; to prepare this paste with a very high concentration of formaldehyd, give it the proper consistence, and fill it in small tin tubes which can be hermetically closed. These tubes I gave to the depots to be sold at the usual cost of making. The large percentage of thymol is given to the paste to insure the permanent sterilization of the pulp fragments. A series of experiments upon pulps of different animals has shown that thymol penetrates the tissue of the pulp very slowly—viz., in the course of several weeks—from one end to the other, and that it retains its antiseptic power, as it seems, for an unlimited time. The formalin, with its rapid action, may soon evaporate and disappear, whereas thymol will stay and be a reliable antiseptic agent. A small piece of the paste is pressed out of tube, brought into the pulp-chamber with an amalgam plugger, and pressed gently against the root stumps of the pulp with a piece of clean cotton wool. The whole operation is done in five minutes; there is no pain inflicted on the patient, and, as far as my four years' experience goes, the tooth will give perfect rest. When the patient comes back to you the next day, or later, the tooth will not show any sensitiveness upon tapping with a steel instrument, a symptom which is nearly always observed after cleaning out the canals and filling the roots. If the inflammation of the pulp has been associated with a slight pericementitis (the pericemental irritation will disappear the day after the ap plication of the formaldehyd paste.

After this method many thousands of cases were successfully treated last year in Germany and Austria, and I feel now so sure of success that whenever a failure in this treatment occurs in my clinic—maybe a patient comes back with pain and pericementitis—I say to my students there has been made a mistake in the diagnosis. Of course, all cases of decomposed pulp, likewise all cases of destruction of the pulp-tissue by pus-formation, have decidedly to be excluded from this treatment. In these cases the canals have to be cleaned out and filled with antiseptics.

For the treatment of septic cases I have recommended for the last two years to my students and to my German colleagues the sulfuric acid process of Dr. Callahan of Cincinnati. I consider this method the greatest progress that has ever been made in the treatment of gangrena pulpæ. After a few applications of the acid the calcific depositions, which we find in almost every case of diseased pulp blocking the root-canals, are decalcified; the canals get wide, and their sterilization has become an easy thing. Neutralization is done with bicarbonate of sodium, or, better, with peroxid of sodium. When the canals are clean, I fill them with the formaldehyd paste above described. Mix a little piece of the paste with a drop of the ten per cent. solution of formaldehyl; and fill the canal with this thin cream. The disinfecting power of the formaldehyd gas that is developing after the introduction of the paste is surpassed, according to my experience, by no other antiseptic agent. I always order the patient to come to a second sitting, though as a rule the canals are sterilized after the first treatment. Very rarely a third sitting is required.

The formaldehyd paste remains in all septic cases as a definitive filling in the canals. It seems to me necessary to fill the roots in septic cases with a soft paste that will never get hard, so that if the tooth should give trouble later on you may any moment enter the canals again with your nerve instrument. And for this purpose the formaldehyd paste seems to be an ideal material.—*Dental Cosmos.*

